# Stigma toward small babies and their mothers in Ghana: A study of the experiences of postpartum women living with HIV

**DOI:** 10.1371/journal.pone.0239310

**Published:** 2020-10-16

**Authors:** Kwame S. Sakyi, Margaret Y. Lartey, Caitlin E. Kennedy, Julie A. Denison, Emma Sacks, Prince G. Owusu, Emily A. Hurley, Luke C. Mullany, Pamela J. Surkan

**Affiliations:** 1 Center for Learning and Childhood Development-Ghana, Accra, Ghana; 2 Department of Public and Environmental Wellness, School of Health Sciences, Oakland University, Rochester, MI, United States of America; 3 Department of International Health, Johns Hopkins Bloomberg School of Public Health, Baltimore, MD, United States of America; 4 Department of Medicine & Therapeutics, CHS, University of Ghana School of Medicine & Dentistry, Accra, Ghana; 5 Health Services and Outcomes Research, Children’s Mercy, Kansas City, Missouri, United States of America; University of Zurich, SWITZERLAND

## Abstract

Infants born to HIV-infected mothers are more likely to be low birthweight (LBW) than other infants, a condition that is stigmatized in many settings worldwide, including sub-Saharan Africa. Few studies have characterized the social-cultural context and response to LBW stigma among mothers in sub-Saharan Africa or explored the views of women living with HIV (WLHIV) on the causes of LBW. We purposively sampled thirty postpartum WLHIV, who had given birth to either LBW or normal birthweight infants, from two tertiary hospitals in Accra, Ghana. Using semi-structured interviews, we explored women’s understanding of the etiology of LBW, and their experiences of caring for a LBW infant. Interviews were analyzed using interpretive phenomenology. Mothers assessed their babies' smallness based on the baby's size, not hospital-recorded birthweight. Several participants explained that severe depression and a loss of appetite, linked to stigma following an HIV diagnosis during pregnancy, contributed to infants being born LBW. Women with small babies also experienced stigma due to the newborns' "undesirable" physical features and other people's unfamiliarity with their size. Consequently, mothers experienced blame, reluctance showing the baby to others, and social gossip. As a result of this stigma, women reported self-isolation and depressive symptoms. These experiences were layered on the burden of healthcare and infant feeding costs for LBW infants. LBW stigma appeared to attenuate with increased infant weight gain. A few of the women also did not breastfeed because they thought their baby's small size indicated pediatric HIV infection. Among WLHIV in urban areas in Ghana, mother and LBW infants may experience LBW-related stigma. A multi-component intervention that includes reducing LBW incidence, treating antenatal depression, providing psychosocial support after a LBW birth, and increasing LBW infants’ weight gain are critically needed.

## Introduction

Low birth weight (defined as weighing <2500g at birth), caused by preterm birth (<37weeks) or intrauterine growth restriction [1], is a leading cause of neonatal death globally [2]. About 15% of the global burden of LBW is in sub-Saharan Africa [3]. In Ghana, 14% of the 776,000 estimated births per year [4, 5] are LBW.

LBW can have significant negative effects on child development, caregiver’s health, and family socioeconomic wellbeing. Short- and long-term mild to severe morbidity and disability, including growth failure, stunting, respiratory problems, and mental illness, are more prevalent among LBW compared to normal birthweight (NBW) [6–8] infants. Caregivers can experience a sense of guilt, self-blame, alienation, prolonged hospital stay, and high levels of psychological difficulties (including depression and anxiety) [9–12]. In one study in Ghana, about 75% of mothers with LBW, preterm, and sick infants reported mild to severe symptoms of postpartum depression [13]. Families of LBW/preterm babies in Ghana spend about 8% of their annual income on their care [14]. Caring for a LBW infant in Ghana affects maternal postpartum HIV care practices, including adherence to antiretroviral therapy and retention in clinical care [15].

HIV significantly increases the risk of LBW in sub-Saharan Africa [16]. In a meta-analysis of 17 prospective studies, 12 (70%) of which were conducted in sub-Saharan Africa, neonates born to mothers living with HIV were twice as likely to be LBW as compared to those born to HIV-uninfected mothers [16]. In Ghana, the odds of having a LBW baby is six times higher for mothers who are HIV-infected compared to those who are uninfected [17]. While much is known about the epidemiology of HIV as a risk factor for LBW [18], women's views on the association between HIV and LBW, and the underlying socio-cultural structures surrounding it, have rarely been explored.

LBW and preterm birth are stigmatized conditions across a number of cultures. In sub-Saharan Africa, a study in Uganda found that some caregivers of small preterm babies rejected their newborns [19]. In one part of Asia, where children with imperfect bodies are stigmatized, some Persian mothers hid their babies, feeling ashamed to show the newborn to their families or in public [20]. Research conducted in Korea identified negative stereotypes about the infants' future mental abilities as a source of stigma toward mothers with LBW/ preterm babies [21, 22]. Commonalities of these studies across cultures include negative stereotypes that exist with respect to physical attributes of small babies as well as maternal distancing from a small baby. In *Death Without Weeping*, in northeast Brazil [23], Scheper-Hughes theorized that in regions with high child death expectancy and poverty, a baby's small size is one marker used to differentiate infants who will survive and thrive from those who are more likely to die. Notably, stigma toward LBW babies may have consequences for child survival, as it may contribute to intentional or unintentional neglect or abandonment [23].

To tackle the adverse effects of stigma, Parker encourages attention to the structural factors that produce stigmatization [24]. In Ghana, LBW disproportionately affects women who are poor, young, unmarried, infected with HIV, have poor nutritional status, and have inadequate access to healthcare [17, 25–28]. These risk factors are shaped by the political economy and women's position in society.

To date, there is a paucity of research on stigma related to a mother's experience of having a small baby in sub-Saharan Africa. Most studies have devoted attention to the care [29, 30], risk factors [27, 31], mortality [2, 32], and maternal mental health [13, 33] associated with LBW infants. In the few studies that describe stigmatizing behaviors [19, 34, 35], stigma has not been a key focus. Therefore, the social-cultural context, nature, and responses to LBW stigma have not been well characterized.

In this paper, we examine the source and nature of LBW stigma, as well as postpartum women living with HIV (WLHIV)’s responses to the stigma. We also present WLHIV’s views on causes of LBW and provide a broader perspective on the lived experiences of WLHIV with LBW infants in Ghana.

## Methods

### Setting

This study was carried out in the Greater Accra Region, the second most populated of the sixteen administrative regions in Ghana. In Ghana, HIV prevalence is 2.0% overall and is 2.8% among women of reproductive age [5]. In the Greater Accra Region, the highest HIV prevalence is among women of reproductive age (15–49 years), at 3.8% [5]. Based on the 2014 Demographic Health Survey, about 5% and 11% of mothers in Ghana perceive their most recent baby’s size at birth to be very small and small, respectively [5].

The study was conducted at two tertiary hospitals, Korle Bu Teaching Hospital and Ridge Regional Hospital, located in the city of Accra, Ghana's capital. Twenty-three thousand deliveries a year occur at these two hospitals. Facility records for 2015 show the prevalence of LBW among WLHIV is about 8%.

### Participants, sampling, and recruitment

We used data from a larger qualitative study that examined how caring for LBW infants influenced maternal adherence to antiretroviral therapy and retention in HIV care in the postpartum period. Postpartum women were eligible for participation if they were living with HIV, gave birth at or were receiving HIV treatment at one of the two hospitals, were 18 years or older, and had an infant under one year of age. Mothers who delivered at home were excluded. Participants included 30 postpartum WLHIV, 15 with LBW infants, and 15 with normal birthweight (NBW) infants (≥2500g), based on hospital-recorded weights at birth. Data were collected from February through April 2016. The first author and two trained research assistants (one male and one female) conducted semi-structured interviews with study participants.

Participants were purposively sampled based on hospital-recorded birth weights of <2500g for the LBW infants, and ≥2500g for the NBW infants. Mothers were recruited via health workers in the maternity ward, adult and pediatric HIV treatment centers, and the neonatal intensive care unit (NICU). Health workers were given a detailed description of the study’s purpose and eligibility criteria. They referred patients who expressed interest in the study to the research team, who reassessed eligibility and obtained written consent.

Using previously pilot-tested semi-structured interview guides, published elsewhere [15], we conducted twenty-eight interviews in Twi and two in Ga. On average, the interviews lasted 46 minutes and were conducted either at the hospital or in participants' homes. The interviews elicited detailed stories about caregivers' thoughts and feelings after their child’s birth, experiences with caring for their babies, situations or context that affected their experiences, and views on infants born small. Interviews were audio-recorded, except for four participants who declined to be voice recorded. In these cases, handwritten notes were taken and transcribed immediately following the interview. All audio-recorded interviews were translated and transcribed into English. After the interviews, participants also completed an interviewer-administered structured questionnaire that included information about socio-demographic characteristics, HIV history, and birth outcomes. Birthweight data were extracted from hospital birth records.

### Analysis

We adapted Smiths' methods for interpretative phenomenology to analyze the data [36]. We selected phenomenology over other qualitative methodologies because it provides a philosophical and theoretical justification to examine lived experience [37, 38]. Phenomenology privileges human consciousness and focuses on how things appear to individuals going through the same situation or experiencing the same condition. Of key interest to the research team were the physical, social, and cultural spaces in which LBW stigma took place. Thus, interpretive phenomenology was selected because it emphasizes the meaning and context (*lifeworld)* of individual experiences [36].

The data collection team first read and summarized each interview transcript to get a global sense of the text. The first and sixth authors inductively developed a codebook. The first author then systematically applied it to selected meaning units, which are significant sentences or phrases related to the phenomenon under study. Coded excerpts were organized and clustered into themes based on similarities and analyzed interpretively. The interpretive process entailed attention to local terminologies and metaphors, and a focus on the temporal construction of narratives [36]. During the analysis, memos were kept related to researchers’ feelings and reactions to the interviews as well as prior and evolving assumptions [39].

### Ethics

All study procedures and protocols were approved by three ethical review boards 1) the Johns Hopkins Bloomberg School of Public Health Institutional Review Board (IRB No. 6651), 2) the Ethical and Protocol Review Boards of the Ghana Health Services (ID: Ms-Et/M.2-P4.1/2015-2016) and 3) the University of Ghana Medical School (ID: GHS-ERC 16/09/15). All participants provided written informed consent. Each participant received the equivalent of $5 and one pack of baby diapers.

## Results

A description of all 30 participants in the study has been published elsewhere [15]. The fifteen participants with LBW infants were on average 34 years of age, had been living with HIV for four years, and had one child. The mean LBW was 1.96kg [range: 1.00kg-2.48kg] (**See Table 1**). Eleven of the 15 mothers with LBW infants considered their babies' birth size as small or very small, independent of the hospital recorded birthweight. On average, infants were six months old at the time of the interview [range: 5 days to 12 months] (**See Table 1)**. The key themes and illustrative quotes from the qualitative interviews are presented below.

**Table 1:** Background characteristics of study participants with low and normal birthweight infants, N = 30.

Characteristics	Mothers with LBW infants, N = 15	Mothers with NBW infants, N = 15
**Categorical Variables**		
**Education level+**		
Primary school or less	6	3
> Primary School	8	11
**Marital status**		
Married/cohabiting	14	15
Single	1	0
**Occupation**		
Trader	7	7
Seamstress	4	5
Other	4	3
**Time of HIV diagnosis**		
Before index pregnancy	13	8
Index pregnancy	2	7
**Continuous Variables**	**Mean (range)**	**Mean (range)**
Maternal age (years)	33.86 (24–42)	35.73 (26–44)
Number of children	1.24 (1–5)	2.35 (1–5)
Years living with HIV	4.31 (0.58–15)	4.25 (0.5–16)
Mean birth weight (kg)	1.96 (1–2.4)	3.01 (2.5–3.5)
Infant age (months)	5.98 (0.5–12)	3.04 (0.2–11)

+ missing data.

### Maternal understanding of LBW

#### “Adwen, adwen–thinking too much” how depression and poor nutrition lead to LBW

In our sample, some participants believed that having a LBW infant was a result of HIV, while others did not. Both mothers with NBW and LBW explained that HIV diagnosis during pregnancy contributed to LBW indirectly through depression and poor nutrition, and directly through infant infection. One participant stated: “*When I first saw him*, *I thought because of how small he was*, *he might be infected with the virus*.*”[P09]* Some women described that HIV diagnosis is often followed by *adwen adwen ("thinking too much" or "excessive worrying or rumination")*. *Adwen adwen*, or its derivative, *dwen dwen*, is a common idiom used in Ghana by clinically depressed patients [40]. Some participants said that the *adwen adwen* leads to having a LBW baby because it results in a loss of appetite during pregnancy. The stories the mothers related suggest that depression and poor nutritional status were primarily connected to either perceived or anticipated HIV-related stigma. Sources of the *adwen adwen* were often related to fear of early death, experiencing public stigma toward WLHIV, accusations of infidelity, mother-to-child transmission, and being separated from a partner. For two women, the *adwen adwen* was severe enough that during pregnancy they either had planned to commit suicide or terminate the pregnancy. One of them stated:

“*Actually*, *it was during my pregnancy that I was diagnosed with this condition [HIV]*, *which greatly affected me*. *I lost the desire to eat*. *I was filled with* adwen adwen. *I think my lack of eating and the* adwen adwen *had an impact on my baby*… *When you hear that you are infected for the first time*, *it is not easy because you think about what may have went wrong*…*I am a wedded wife*, *too*. *I wanted to abort the baby because I was afraid that I might infect the baby*.*” [P31]*

Several mothers did not believe that HIV contributed to having a LBW birth, citing instead causes including birth history, maternal illness during pregnancy (e.g. frequent diarrhea, and excessive vomiting), prematurity, infant illness at birth, *asram* (a local illness thought to be caused by evil eye), and inadequate nutrition (e.g. *We concluded that the woman and the baby were not well fed that is why the baby’s weight was low*.” [P11]) as causal factors. Participants also observed that not all of the babies they saw at the NICU were born to WLHIV. As one mother stated: “*Some of the mothers at the NICU were not infected with this illness*, *but they also gave birth to small babies*. *What about that*?*" [P32]*. Maternal illness, particularly excessive vomiting during pregnancy, and preterm birth were the most common non-HIV factor discussed.

#### *Kete kete* (tiny, tiny): Mothers’ language for LBW infants

Participants based their assessments of their babies' smallness not on the hospital-recorded weight but rather on the baby's appearance. Mothers commonly used three phrases to describe the size of their babies at birth: 1) "*Na wosa paa*" (the child was really small), 2) "*Na wo ye ketewoa bi" (*the child was tiny*)*, and 3) *Na wo ye kete kete (*the child was tiny tiny)." Some babies were LBW, but their mothers did not consider them *kete kete*. Other names included the English word “premature (the most common),” *"aketew"* (the tiny one), "*obumpa*" (sarcastically, the one who is heavy enough to break a bed)," *"kukuba"* (the baby born small enough to fit into a pot) and “*Asram kotrɛ”* (an illness that makes a baby as small as a lizard). Designation as being small did not always occur at the time of birth. Some participants with NBW babies indicated that their babies were born “normal” or “big” but became small later because they lost weight.

None of the mothers with small babies, except one, associated a baby’s size with a long-term disability; however, the majority seemed to be aware of the poor chances for survival of LBW infants. Mothers who described their babies as small, compared to those who did not, reported more fear and uncertainty about their infants' survival because of their size, particularly among those who were hospitalized at the NICU. As one stated: *“Any time I went to the NICU*, *I did not feel good*. *Sometimes*, *I felt they would tell me the child has died*.” [P32]).

### LBW stigma toward mother-infant dyad

**Fig 1:**
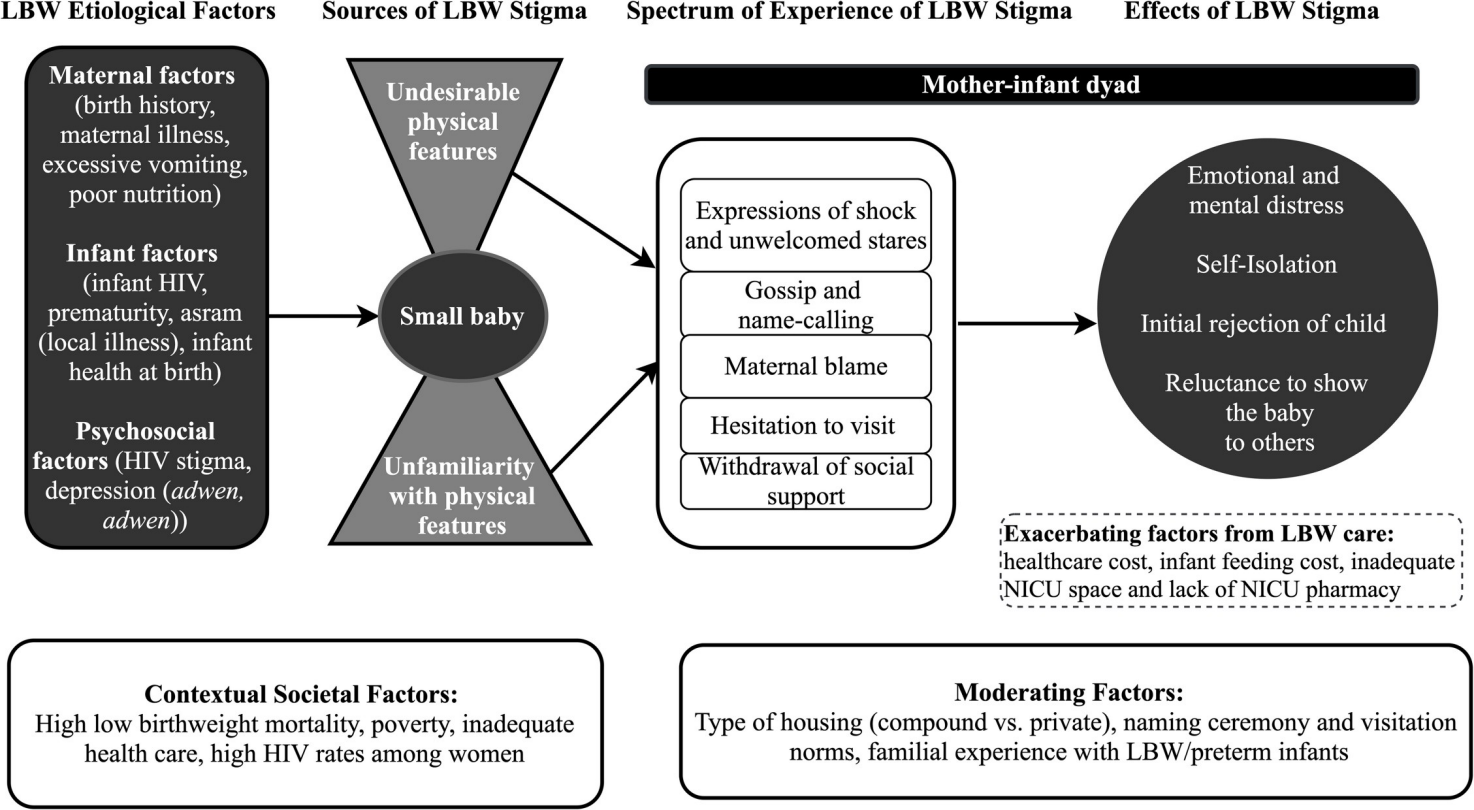
Conceptual model of LBW stigma toward the mother-infant dyad: The experiences of women living with HIV in Ghana.

While none of the WLHIV with NBW babies reported experiences or expectations of stigma relating to their baby’s size, about half of the participants with LBW infants did. Relying on the women’s accounts, we describe the sources, spectrum of experiences, effects, and management of LBW stigma below. We illustrate the relationships between these dimensions of stigma in **Fig 1**. It is important to emphasize that several participants did not report experiencing negative treatment from individuals within their family and social circles. Among these participants, many said their family members or friends had seen a small baby before. One stated: “*Most of them had seen a premature baby before and had positive views about their future outcomes*. *[P33]*” Some did not let others see their baby to avoid social stigma. One group that mothers felt did not stigmatize them were healthcare workers at the NICU. All the participants who had children admitted to the NICU praised the healthcare staff. They felt that the staff made them feel "*like human beings"* and did not *"shout or yell"* at them. They also called their *"baby by their [mother’s] name* " and treated the babies "*as if they were adults*."

#### Sources of LBW stigma

Most participant narratives indicated that stigma toward small babies and their mothers was closely connected to the babies' 'undesirable' physical features and people's unfamiliarity with the baby's size. These two factors led to what Goffman has called a "spoiled" identity [41]. Several participants compared the baby to an animal (commonly lizards, frogs), and others emphasized how physical appearance of small babies looked 'abnormal' compared to other newborns.

"*I said 'No—that could not be my baby*, *and that it was an animal*, *and I cannot believe that I have given birth to such a baby*.*'…There were some of the mothers sitting outside*, *and they were talking about me*.*" [P08]*

Despite this negative characterization, in a few cases, there were also positive reactions—that LBW infants are resilient and could overcome their low weight and reduced chances of survival and become "*fatter*" or *"stronger"* over time, if they are well-nurtured.

#### Spectrum of experience of LBW stigma

Some mothers reported that the attributes of their baby resulted in negative social interactions toward them and their child. A wide spectrum of reactions discussed ranged from an expression of surprise to overt restriction of social support to the mother-infant dyad. Women seemed to feel that society saw them as responsible for the birth of such a small child, as illustrated in this quote:

" *She [my sister] asked me*, *'Why have you brought such a small into this house*? *You are always pointing out the flaws in others' children and insulting them*, *have you seen what you have given birth to*? *Is this what you have given birth to*?*'… I told her to leave me alone*.*" [P06]*

Women also said that their friends or neighbors sometimes refused to visit mothers with small babies. For example, "*In this modern Ghana*, *when you have such a child*, *people would not to come around you and your child*. *[P01]”* Gossip, name-calling, and unwelcomed stares were experienced by some of the participants. For example, one woman described how people close to her home would call her “kangaroo” (a reference to the well-known “kangaroo mother care (skin-to-skin)” method for caring for LBW infants) as an insult: "*When I used to go to the NICU*, *I would tie her [the small baby] on my back*, *and so when people saw us coming*, *they would yell*: *"kangaroo" is coming*.*" [P19]*

Another example of social stigmatization is the experience of maternal blame perpetuated by other mothers or non-NICU healthcare workers:

"*Oh*! *They [women at the postnatal clinic] also stare*. *It's not different*. *They really stare at you*!…*when I [also] went there [postnatal clinic]*, *they [nurses] blamed me that I was not giving the child breast milk [and] that is why the baby looked the way it did*.*" [P25]*

One participant’s secondary account suggests that expectations from family members, along with stigma from others, constrained the choices of mothers of very small babies and contributed to child abandonment, although mothers resisted this pressure.

"*Stigmatization goes with preterm delivery*… *Some people can advise the mother to terminate the child's life… Some people*, *if they do not terminate the child's life*, *they will abandon the child*…*Some cannot withstand the social pressure*. *For example*, *a teenage girl had this preterm baby*, *and her sister advised her that she should strangle her*, *but the mother did not have the courage to do so*. *They then quietly abandoned the baby [at the hospital]*. *People can say*: *"Go and dump the baby in the bush or by the riverside because the baby is too tiny*…*They are afraid*. *They feel they have invested so much into the pregnancy—the time*, *the stress*, *and the resources*. *The expectations they have*, *you know*, *have been interrupted… Some of them are afraid of the stigmatization—If I send this child home*, *what will my mother- or father-in-law say*? *What will society say*? *Will they accept the baby*? *Will my husband understand the situation*?*" [P11]*

### Effects of LBW stigma: Emotional distress, psychological challenges and self-isolation

Mothers reported experiencing emotional and mental distress due to others' reactions to their baby’s size and appearance. Some mothers felt discouraged and embarrassed, and others experienced depression-related symptoms, prolonged sadness, crying all the time, and *adwen adwen* [40]. For example, one participant recalled:

*My husband's sister*… *used to encourage me…but the members of the house made me feel discouraged…I used to cry when I was alone with my child*…*[P17*]

Another effect was self-imposed social isolation of the mother-infant dyad that emanated from the reluctance to show the baby to others (described earlier), which also limited the child’s interaction with others. In contrast, interviews with mothers with NBW babies suggest that infants had more family members and neighbors interact with their children. Moreover, the social pressure some mothers faced (discussed earlier) unduly contributed to child abandonment. Maternal rejection of the infant was observed in one interview: “*I told the nurse that this animal was not my baby because when I give birth*, *my babies are beautiful—and why is this one like this*? *I told them to look for my baby*.*” [P08]*

It is essential to point out that, the emotional, social, and psychological effects were similar to those that many women described they faced at the NICU, because of the poor survival chances of LBW infants and the limited control they had over their child’s care at the NICU.

“*Oh*!, *she was very small*. *I did not even know she would live*. *[It] made me unhappy because I wondered if she would make it*.*” [P18]*

“*They do not allow you to come close [to some of the babies]*. *Every day*, *the baby would cry [at the NICU] and you could not do anything*. *[Sometimes] you just went there and watched [through the doors] because you [generally] heard a baby cry*. *As a mother*, *you feel something*, *like a sting*, *inside of you*, *but you cannot do anything*… *Sometimes you do not see your child with your own eyes*. *You feel unhappy*. *You feel dull*. *You feel fear*…*You do not want the baby to die*!*” [P27]*

Mothers with NBW infants did not report emotional and psychological difficulties based on their infants’ size.

### Effects of LBW stigma: Reluctance to show the baby to others

Most mothers with small babies were very reluctant to show the baby to others. In contrast to mothers with NBW infants, mothers with LBW infants reported using various strategies to prevent others from seeing their infants because of their size and appearance. These strategies included making excuses to prevent others from visiting them, hiding their babies before a visit, avoiding public transportation or covering the babies, and delaying the “outdooring” of the infant, a naming ceremony in which the child is symbolically given an identity as a human being [42]. The participant who received the stares at the postnatal clinic recalled her experience at home:

“*One day*, *we were outside of the room to get some fresh air*, *and I didn't know that she [a family friend] was coming to visit me*. *When she made it closer to the house*, *I quickly took the baby inside*. *I told her that she could not visit because we had not officially "outdoored" the baby*…*It was not until we did the outdooring that we let our neighbors see the baby*. *We were able to bring him out because he looked much better than when he was born*.*” [P25]*

One emerging theme was that women's socioeconomic status strongly influenced their ability to regulate people's exposure to their child. Mothers who lived in a single-family housing (such as their own home or apartment) were able to control when and how others saw their child more so than women who lived in a multi-family compound house, which has a shared courtyard and amenities [43]. A compound house is typically inhabited by low-income families. Given the shared courtyard and multiple families, women described how it took more careful planning to prevent neighbors from seeing the child in a compound house, particularly during bathing "*…I waited until no one was around before I bathed her…[because] they would have said that the child was small and all that" [P18]*. One mother who did not want to keep her baby in-doors or regulate her childcare practices in her compound house suffered the worst social mistreatment of all the participants in the study. She was labeled a witch, experienced withdrawal of social support, gossip, and pressure to leave her home. She also felt her neighbors might have known her HIV status.

“*They said the child was too small and that I should take her to my mom [which she later did]…The child cried very loudly too often*, *so when I bathed her [in the compound]*, *people would approach*, *and see how tiny she was*. *They thought I was a witch… The child was small [so] they used to whisper among themselves… They might have heard that I had gotten this illness [HIV]*…*[because] the person who was supposed to help me bathe the baby was later reluctant to do so*… *As for my child*, *I brought her outside early since she did not like to stay indoors…” [P17]*

#### Management of LBW stigma

The strategies used to endure stigma and manage the broader psychosocial challenges associated with the care for LBW infants varied across participants. These strategies included regulating visitations, seeking social support for feeding and encouragement from others, disregarding negative comments, resisting maternal blame, and recognizing that there were others with LBW babies facing similar or even more difficult challenges. For example,

“*At that place (NICU)*, *when you see other people's babies*, *you will be thankful for your baby; even those parents [with smaller and sicker children] …are [finding reasons to be] thankful*, *how much more me*?*” [P30]*

“*You cannot question me*! *I did not hide the baby because the baby was full term*. *He was nine months old when he came out*. *I cannot throw the baby away*, *so why would I be worried about what people think about his smallness*?… *Is it compulsory that the baby has to be healthy*?… *Other kids are big*, *but they are not healthy*.*” [P27]*

One observation from the data was that the negative psychosocial effects related to LBW infant care and stigma were attenuated with adequate infant weight gain, an outcome that consistently resulted in relief and pride. One of the participants who had experienced severe *adwen adwen* described her daughter who weighed 1.2kg at birth:

“*She had really put on weight by the fourth month… When we go to the clinic now*, *the doctors and nurses all run to receive her because they know she was a premature baby*. *They all get excited because they know I have taken good care of her* [*participant smiles and shows interviewer her baby's picture]*.*” [P31]*

### Exacerbating effects of LBW care

Stigma was not the only lived experience of WLHIV with LBW infants. Care of LBW infants also required financial investments from families. Hospital admission-related costs were the major, but not the only, source of economic strain. Limited resources at the NICU compounded the burden. Mothers said that they paid for the number of days the baby stayed in the NICU or general pediatric wards. One woman explained: “*At Korle Bu*, *even if you have health insurance*, *after you are discharged*, *they will give you a bill to pay*.*" [P09]*. The NICU also did not have space for mothers to stay overnight with their babies. Consequently, some mothers with financial means paid for accommodation and food on-site. Others went home and paid for the commute. As one mother pointed out, some of those who could not afford their hospital costs do not return. Moreover, the two hospitals also did not have a pharmacy at the NICU, so mothers had to locate and purchase prescribed medications elsewhere.

“*The children of some of the mothers are admitted to the NICU for a long time… [but] they do not have a place to sleep*. *Some [women]*, *too*, *prefer to go home and come back [the next day] since they are unable to pay the ward bills…Some [who are unable to pay] even do not come back because the NICU will still take good care of the baby if they do not…The food I bought from the NICU was quite expensive*. *It was 40 Ghana Cedi (~ U*.*S*. *$10) [a day]…The place [NICU] also does not have a pharmacy…I had to go the suburbs of Accra to find some of the prescribed medications*. *It is not good for a mother who has just given birth to roam in search of a medication*. *Women who have had a cesarean section*, *upon their return*, *sometimes collapse at the NICU because of the stress they had to go through*.*” [P23]*

Another source of economic stress was buying infant formula. Desire to prevent mother to child transmission of HIV was the most common reason for needing formula, but a few of the women also did not breastfeed or weaned early because they thought their baby's small size suggested that the child was already infected with HIV.

“*It was there [at the hospital] that I decided not to breastfeed*, *but use baby formula instead…I did not want her to be infected through the breastmilk since the virus may be in my blood… Every nursing mother would testify that it [the cost] is a problem*. *I just even called someone for money to buy food for her because she will be running out of food soon*. *Just one can of baby food costs GHC 25 (~$5)*.*” [P19]*

“*I initially wanted to breastfeed him*, *but because of his small size*, *I thought he was infected [with HIV]; I weaned him from breastfeeding as a result [in order not to give him more of the virus]…My other children even breastfed for more than a year*.*” [P05]*

## Discussion

This study is the first to examine lived experiences of WLHIV with LBW infants in a sub-Saharan African context. Their experiences and perceptions of LBW reveal the sociocultural context of stigma toward LBW babies, the structural context that shapes it, and the burden that stigma places on individual mothers and their infants. Collectively, mothers attributed LBW to varied and nuanced causes or influences, including maternal (prior birth history, maternal illness, depression, nutrition), infant (HIV infection, preterm birth), and social (HIV stigma) factors. Participants reported significant negative consequences of LBW for both maternal and infant wellbeing, including maternal depression and isolation, which compounded the economic and psychological difficulties associated with NICU care. Women in the study demonstrated agency by resisting blame and leveraging social and cultural norms to avoid enacted stigma.

Both Lupton and Merleu-Ponty's discourse on interembodiment, the idea that primary caregivers (such as a mother) and their infants' bodies are intertwined, argue that through pregnancy and childcare practices, the experience of a mother is inseparable from that of the infant [44, 45]. Although they appear autonomous, their bodies and lives inevitably shape each other and exist in relation to each other. Thus, we can conceptualize LBW stigma as a lived experience of the mother-infant dyad, rather than as an experience of the child [19] or mother alone [20]. In this study, for example, we observed that the source of stigma toward small babies was the infant's body; however, people also blamed the mother for that outcome. In addition, the mother-infant dyad suffered loss of social status as a unit. An illustration of this was the delay we observed in families’ outdooring their small babies. The Ghanaian naming ceremonies bestow a "human" identity on the infant and celebrate the maternal body for giving birth. Status loss is consistent with recent conceptualizations of stigma [46]. Moreover, mothers’ attempts to prevent others from seeing their babies’ bodies, as a means of reducing their own experience of stigma, also contributed to prolonged isolation of the child from others.

Based on our findings, the nature of social stigma towards small babies in Ghana appears to be distinct from other settings, in that it is not tied to their future capabilities and productivity. In contrast, a Korean study found that the source of stigma toward LBW infants was more related to children's potential future mental disability than appearance [21]. However, in our study, consistent with reports by others in Ghana [35], we did not find a prominent perceived association between a baby's size and future mental or physical disabilities. In fact, mothers' narratives suggested that one way that others encouraged them was to highlight how such children would become intelligent, healthy, and productive in the future. One possible explanation for the differences across settings is that in places with very high mortality in children, such as in Ghana, social stigmatization around a baby's size is organized around survival goals [23]. According to this theory, attitudes toward the baby are likely to change from negative to positive once the child survives past high-risk periods or show signs of thriving [23]. This interpretation is consistent with our findings in Ghana which shows that stigma attenuated with a baby's weight gain.

Stigma is inherently connected to structural violence, or the disadvantages and harm that social, economic and political systems place on certain groups [47, 48]. LBW is estimated to account for about 60%-80% of all neonatal deaths [49]. Despite increasing evidence for low-cost, effective infant survival practices, incidence in Ghana has remained about the same for the past decade [5], suggesting structural barriers. Food insecurity can compromise maternal nutrition and increase risk of LBW births, particularly among WLHIV [50]. Poverty, low quality health care, and neighborhood deprivation are major structural causes of deaths among LBW neonates in Ghana [5, 28, 51, 52]. The reluctance to visit a LBW baby may also be linked to their high mortality rate, and visitors fear of been blamed for their deaths, which is consistent with local beliefs of illness and causes of newborn deaths [53, 54]. Housing and neighborhood characteristics are ways that structural violence confers unequal vulnerability to suffering [55, 56], and women in Ghana have a three-fold increased risk of a neonatal death if they live in a community with high socioeconomic disadvantage [51]. Participants in our study who lived in a single-family, private homes (e.g. higher income) experienced less LBW stigma (albeit increased social isolation) compared to those who lived in a multi-family, low-income compound houses. Overall, structural factors contribute to inequity in the incidence of LBW and to related social and health consequences of LBW.

While other studies have reported psychological and emotional stress mothers bear due to fear that their baby may not survive, our work suggests that the conditions of urban WLHIV may exacerbate and prolong maternal mental health concerns. Previous studies of the experiences of LBW and preterm babies have mostly focused on HIV-uninfected mothers' experiences, particularly in the NICU [10, 33, 35]. They show that mothers of LBW babies experience short and long-term mental health difficulties [13, 14, 35, 57], including depression [58, 59], trauma [60] and stress from the economic burden of LBW infant care [46]. We found that HIV stigma, especially experienced immediately after diagnosis, also played a role. WLHIV in our study commonly experienced the onset of depression at the time of diagnosis (often coinciding with pregnancy) and attributed depression and poor maternal nutrition as causes of LBW. Our unique finding suggests that the effect of HIV-stigma among WLHIV may extend beyond the mother’s wellbeing and engagement in HIV care [61–63], and it may negatively contribute to poor birth outcomes through its effect on depression and poor nutrition.

The results have implications for public health and clinical practice. As part of efforts to reduce the prevalence of LBW and preterm birth, pregnant women should be screened and treated for depression. One meta-analysis with 25,663 women found that untreated antenatal depression increased the odds of LBW and preterm birth by 96% and 56%, respectively [64]. A critical time point to screen pregnant women for depression is at the time of HIV diagnosis, or when WLHIV realize they are pregnant, as anxieties around HIV and pregnancy may be compounded.

Efforts should also be made to reduce stigma related to preterm birth and low birthweight, especially targeting WLHIV. Depressive symptoms and social pressure the study participants faced calls for NICU teams in Ghana to include mental health practitioners. Moreover, linking women to existing mother-to-mother support groups could leverage the experience of mothers who have lived through these challenges to support newer mothers. Finally, LBW stigma and psychological difficulties related to LBW infants’ care among WLHIV in urban areas of Ghana appear to diminish with an infant’s increased weight gain. Thus, nutritional and nurturing care interventions that increase weight gain in LBW infants are recommended.

Our study has several limitations. First, our participants included only women living in an urban area. Further, because all women in our study had HIV, we do not know if these women were also different from the general population in other ways, thus the transferability of our findings to different parts of Ghana is unknown. Future studies should include rural women, relatives of mothers with small babies, and health workers to give a holistic picture of women’s experiences. A strength of our study is that it is one of the few investigations in sub-Saharan Africa that has used qualitative methods to get an in-depth understanding of newborn size as a stigmatized condition. Furthermore, the development of a conceptual model around stigma towards the mother-infant dyad in this setting could serve as a framework for measuring and evaluating the effects of this stigma in Ghana and other similar sub-Saharan Africa settings.

## Conclusion

Being born small at birth is a cause of stigma against mother-infant dyad among WLHIV in urban areas of Ghana. Our findings suggest that LBW stigma in the short term may contribute to poor maternal mental health outcomes and maternal-infant isolation. The findings support a multi-pronged approach, including prevention of LBW, sensitization, counseling, and a multi-disciplinary health care team to address this stigma and its consequences.
